# Targeted Delivery of Genome Editors *in vivo*

**DOI:** 10.1038/s41587-025-02945-w

**Published:** 2026-01-12

**Authors:** Wayne Ngo, Jamie L. Y. Wu, Kevin M. Wasko, Jennifer A. Doudna

**Affiliations:** 1 Innovative Genomics Institute; University of California, Berkeley; Berkeley CA, USA.; 2 Gladstone Institute of Data Science & Biotechnology; San Francisco, CA, USA.; 3 California Institute for Quantitative Biosciences, University of California, Berkeley; Berkeley, CA, USA.; 4 Department of Molecular and Cell Biology, University of California, Berkeley; Berkeley, CA, USA.; 5 Gladstone-UCSF Institute of Genomic Immunology; San Francisco, CA, USA.; 6 Howard Hughes Medical Institute, University of California, Berkeley; Berkeley CA, USA.; 7 Molecular Biophysics and Integrated Bioimaging Division, Lawrence Berkeley National Laboratory; Berkeley, CA, USA.; 8 Department of Chemistry, University of California, Berkeley; Berkeley, CA, USA; 9 Li Ka Shing Center for Genomic Engineering, University of California, Berkeley, Berkeley, CA, USA

## Abstract

Genome editing has revolutionized the treatment of genetic diseases, yet the difficulty of tissue-specific delivery currently limits applications of editing technology. In this review, we discuss preclinical and clinical advances in delivering genome editors with both established and emerging delivery mechanisms. Ultimately, targeted delivery promises to significantly expand the therapeutic applicability of genome editing, moving closer to the ideal of a precise “magic bullet” that safely and effectively treats diverse genetic disorders.

## Introduction

The advent of genome editing has unlocked the possibility of treating genetic diseases at their root cause. A diverse range of genome editing systems, including RNA-guided endonucleases, recombinases, and their engineered derivatives such as base, prime, and epigenetic editors, now enable precise modification of DNA within living cells, offering therapeutic potential across a wide range of conditions. However, the clinical translation of genome editing technologies remains hampered by delivery efficiency. While genome editors can perform precise molecular surgery, they lack intrinsic mechanisms to reach their intended cells in the body. Delivery vehicles such as lipid nanoparticles and viral vectors provide physical transport, but their biodistribution is often broad and non-selective, raising concerns about off-target editing, toxicity and immune responses.

Many of these concerns could be addressed if genome editors were delivered exclusively to specific cell types within the body. In the ideal scenario, genome editors and their delivery vehicles would circulate inertly through the body, bypassing healthy tissues, and accumulate only in the intended cell type. They would carry out their editing function in targeted cells, leaving all other cells unaffected. In theory, such targeted delivery allows therapeutic cargos to be concentrated exclusively in specified tissues, mitigating off-target side effects and immunogenicity by limiting systemic exposure.

Although achieving this level of specificity in the body is challenging, various strategies exist or are in development to limit genome editing to certain cell populations. In this review article, we discuss these targeting mechanisms, examine their capabilities and limitations, and consider how they may be applied to fulfill the promise of targeted delivery.

### What is targeted delivery?

Targeted delivery in medicine refers to methods that direct therapeutic agents to specific cells, tissues or pathogens, while avoiding bystander cells. The concept of specifically targeting pathogenic cells with therapeutic agents emerged in the early 1900s. In 1897, German physician-scientist Paul Ehrlich published the “side-chain theory of immunity”, where he hypothesized that antibodies bind to antigens through special chemical structures he called “side chains”. Ehrlich proposed that the binding of “side chains” to an infectious agent or antigen was specific and analogous to the fit between a lock and key. These insights eventually led Ehrlich to propose the idea of *Zauberkugel*_,_ or “magic bullet”, a hypothetical agent that can kill specific microbes without harming healthy tissues in 1908. Ehrlich postulated that to kill microbes, “wir müssen chemisch zielen lernen” (“we have to learn how to aim chemically”). His efforts led to the discovery of Salvarsan, a small molecule drug, in 1909 for syphilis, credited as the first “magic bullet”. While Salvarsan and its derivatives had side effects, indicating some level of nonspecificity, the drug remained the main treatment for syphilis until antibiotics were developed in the 1940s. Since this initial demonstration, the holy grail of “magic bullets” has persisted as new tools and chemistries, such as antibody-drug conjugates, aptamers, viral vectors, and nanotechnology, expanded our ability to control specificity.

The combination of these tools and chemistries with genome editors theoretically allows controlling specificity at multiple stages of the delivery and editing process. First, researchers can control where genome editors accumulate in the body by modifying genome editors or delivery vehicle properties such as size, chemical properties and the inclusion of targeting ligands that bind to specific cell-surface receptors. These modifications allow for organ- or tissue-level targeting. Second, beyond accumulation, researchers can restrict the expression of genome editors to select cell types, ensuring that genome editing only occurs in cells with the appropriate transcriptional or post-transcriptional environment. Third, specificity can also be imposed at the level of the genetic target itself. By selecting gene targets that are uniquely or preferentially expressed in particular cell types or disease states, researchers ensure that even if the editing machinery is active in multiple cell types, phenotypic changes will only be reflected in the intended target cells. In the following sections, we describe recent *in vivo* genome editing advances categorized by their mechanism of targeting and we discuss their advantages and disadvantages. For comprehensive discussions of *in vivo* gene editing efforts in the clinic, genome editing advances categorized by organs and delivery modalities (see box 1 for a brief comparison), toxicity and immunogenicity of genome editing, we direct readers to other recent reviews.^1–5^

The holy grail of targeted delivery is to achieve exclusive physical accumulation of therapeutic agents in diseased cells without any uptake by healthy or bystander cells. This ideal represents the most stringent form of targeting, akin to throwing billions of darts that only hit bullseyes. Realization of this level of precision in delivery would have profound implications for therapeutic effectiveness and safety. Such precision ensures maximal dosing efficiency. Every molecule of therapeutic cargo contributes to the intended outcome, with no dilution of effect in off-target tissues. This allows for lower overall doses, minimizes manufacturing burden, and improves the therapeutic index of genome editing systems. In addition, this approach minimizes risks associated with exposure to genome editors. By restricting delivery to a defined subset of cells, the potential for systemic toxicity, off-target editing and immunogenicity is reduced.

## Controlling the physical location of enzymes by the administration method

**Figure 1.**
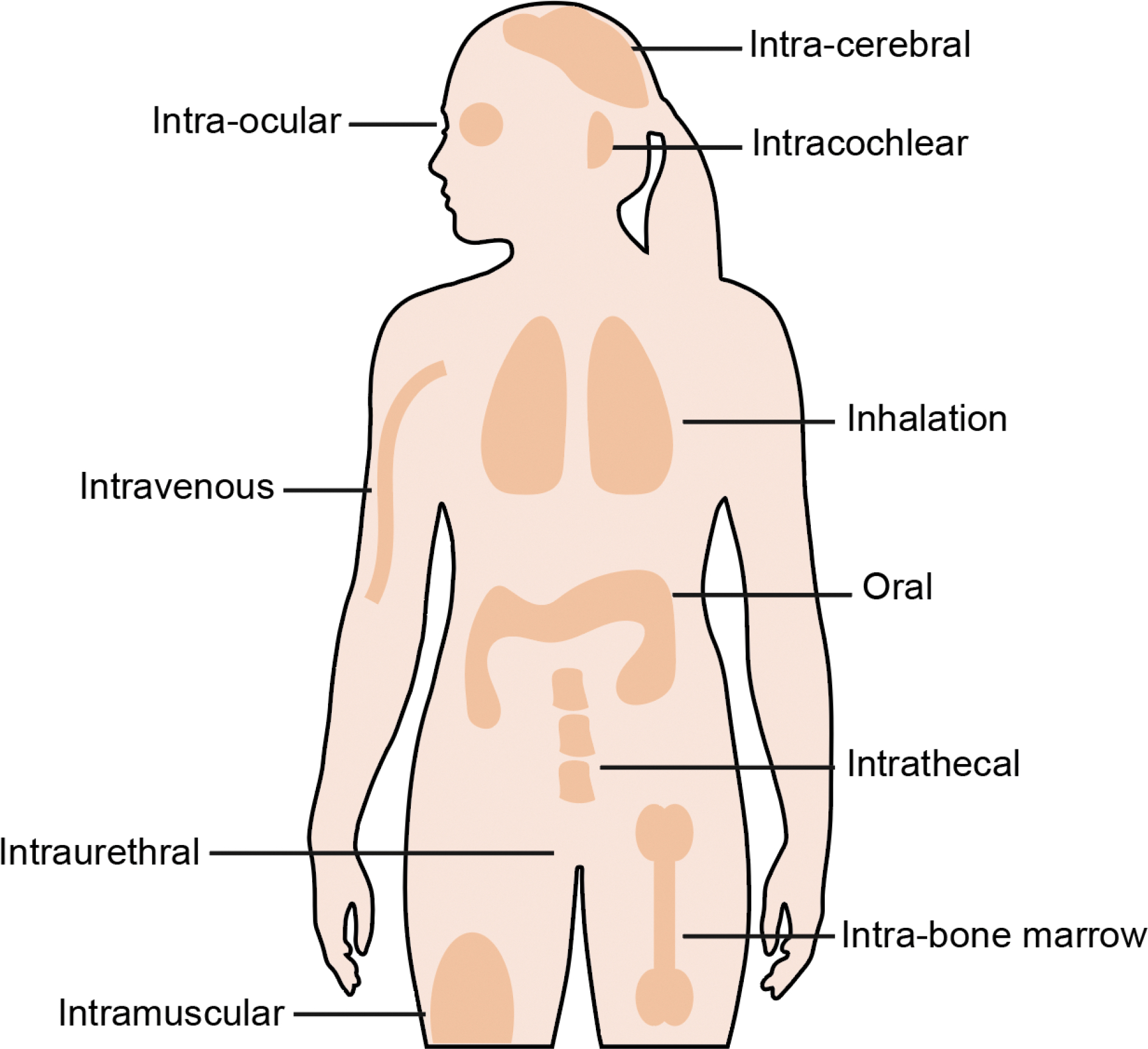
Schematic of administration routes. Schematic not to scale.

The most direct method of controlling the physical organ and tissue distribution of delivery vehicles and cargo is by choice of administration route (Fig. 1). Different administration routes lead to distinct biodistribution and pharmacokinetic profiles. Intravenous administration delivers cargo and delivery vehicles directly in the bloodstream for systemic circulation. Intravenous administration is desirable because it is generally non-invasive, but can result in broad distribution of therapeutics, with clearance organs (such as the liver) taking large fractions of the dose.^6^ This has made intravenous administration especially suitable for genome editing in liver cells. For example, Intellia’s NTLA-2001, a lipid nanoparticle (LNP) formulated with Cas9 mRNA and single-guide RNA (sgRNA), is administered intravenously and capable of reducing baseline transthyretin (TTR) protein levels by up to 87% in patients after 28 days to treat transthyretin amyloidosis.^7^ More recently, an infant was administered two intravenous infusions of LNPs (at 7 and 8 months of age) packaging an adenine base editor (ABE8e) mRNA and sgRNA to treat a pathogenic mutation (Q335X) in the carbamoyl-phosphate synthetase 1 gene. While liver biopsies to assess editing were not performed, the patient had improved biomarker levels and remained neurologically stable.^8^ These therapeutic successes gives hope that similar treatments could be developed for hundreds of hepatic inborn errors of metabolism. Similarly, another LNP-delivered ABE mRNA with sgRNA (VERVE-101) targeting proprotein convertase subtilisin/kexin type 9 (PCSK9) was used to treat heterozygous familial hypercholesterolemia.^9^ Ongoing trials using intravenous administration include treatments for hereditary angioedema^10^, homozygous familial hypercholesterolemia^11^, alpha-1 antitrypsin deficiency^12^, atherosclerotic cardiovascular diseases^13^, Duchenne Muscular Dystrophy^14^, glycogen storage disease type 1^15^, primary hyperoxaluria type 1^16^, hepatitis B^17^, and human immunodeficiency virus.^18,19^ While intravenous injections have been used to treat cells in other tissues, such as muscle^14,20^, the high accumulation of macromolecules and delivery vehicles in the liver can cause potential hepatotoxicity and makes it worthwhile to consider alternative administration routes.

Ideally, agents should be administered as close as possible to target organs and tissues to minimize loss along the delivery journey. The eyes are immuno-privileged organs with specialized barriers that reduce immune response to preserve vision.^21^ The blood-ocular barrier regulates and restricts the passage of macromolecules, viruses and cells into the eye from the blood.^21^ This renders the delivery of agents intravenously into the eye inefficient. The blood-ocular barrier, combined with the lack of lymphatic outflow, may help the eye retain locally administered agents.^22^ Intriguingly a study found that despite the presence of pre-existing anti-Cas9 antibodies in the serum, there were no antibodies in the vitreous of the eye until mice were administered Cas9 intraocularly.^23^ Subretinal injections can be used to inject agents directly into the eye. In subretinal injections, a small volume is injected under the retina into the space adjacent to the photoreceptors. Sub-retinal injections are used in the BRILLIANCE trial for Leber congenital amaurosis type 10 (LCA10). An adeno-associated virus (AAV) with tropism for photoreceptors was used to package and deliver DNA encoding a *Staphylococcus aureus* Cas9 (SaCas9) expressed with the photoreceptor cell-specific *GRK1* promoter and two specific gRNAs against the *CEP290* IVS26 variant. Viral genomes were only detected in the blood of 36% of the patients, suggesting that the agents were retained in the eye.^24^ Nearly half of the participants (6 of 14) showed improvement in cone photoreceptor function as measured by full-field stimulus testing.^24^ Similarly, there is ongoing work using subretinal injections of AAV8 packaging Cas9 enzymes to treat retinitis pigmentosa^25^, AAV9 packaging adenine base editors to treat Stargardt disease^26^ and AAV packaging Cas13 to treat neovascular age-related macular degeneration.^27^ Beyond subretinal injections, it is also possible to introduce genome editors directly into the eye using intracameral injections^25^, intrastromal^28^ and intravitreal injections^29^.

Other routes of administration currently explored in clinical trials include oral administration, intracochlear injections, intracerebroventricular injections, and intraurethral administration. Oral administrations may be suitable for delivering agents to the gastrointestinal tract. SNIPR biome has ongoing clinical trials in which engineered phages (SNIPR001) delivering the type I-E CRISPR-Cas system were administered orally to specifically kill pathogenic and antibiotic-resistant *E. coli* in the gut microbiome.^30,31^ Oral dosing over seven days was well tolerated in 36 healthy individuals and the treatment lowered gut *E.coli* levels.^32^ Intracochlear injections are currently used to administer AAV9 packaging mini-dCas13X RNA base editors to correct hearing loss induced by a mutation in Otoferlin (OTOF).^33,34^ AAV packaging a high-fidelity Cas13Y was administered intracerebroventricularly to knock down methyl-CpG binding protein 2 (MECP2) to treat MECP2 duplication syndrome.^35,36^ Intraurethral administration was used to administer a CRISPR Cas3-enhanced bacteriophage in the ELIMINATE trial to target *E. coli* bacteria causing urinary tract infections.^37,38^ Outside the clinical trials, researchers have explored using intramuscular injection for muscle^39^, intra-bone marrow injections for bone marrow^40^, intratracheal administrations for lung delivery^41^, and intratumoral injections for tumor delivery.^42^

A major limitation of controlling the physical location of cargo and delivery vehicles by administration method is that many cell and tissue targets (e.g., pancreas) do not have direct administration routes, therefore requiring more invasive delivery methods. While proximal administration routes can improve delivery efficiency and retention compared to systemic administration, they are inherently more invasive. This can increase procedural risk, require specialized surgical expertise, and may limit patient acceptance or the feasibility of repeated dosing. It is also important to consider that diffusion of agents within tissues may be limited.^43^ Lastly, administration methods are generally incapable of targeting specific cell types within the tissue. Achieving cellular resolution of delivery necessitates additional targeting strategies, such as molecular targeting.

## Controlling the physical location of enzymes by molecular targeting

**Figure 2.**
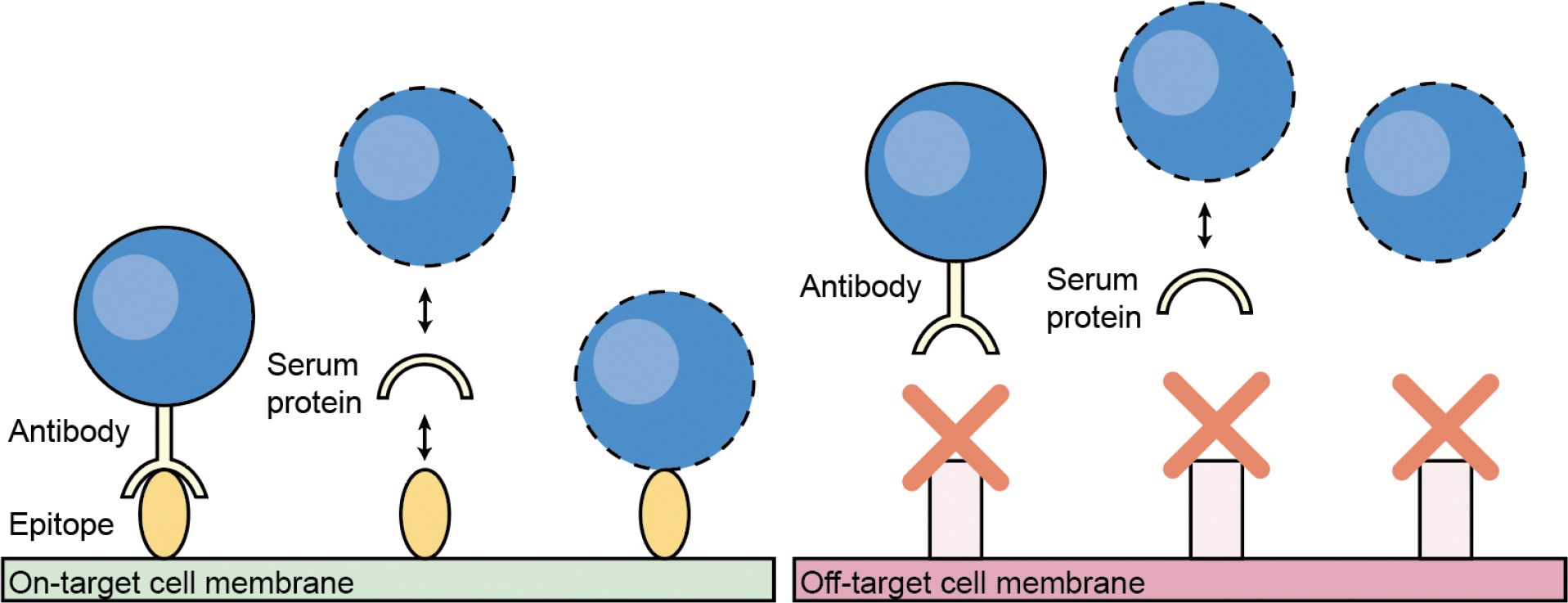
Schematic of molecular targeting strategies, where the goal is to bind delivery vehicles or genome editors to the target cell (left, green) and avoid off-target cells (right, red). Antibodies can be conjugated to the surface of delivery vehicles to mediate binding to specific epitopes on the target cell surface. Alternatively, the chemistry of the delivery vehicle can be modified (dotted lines) to enrich specific proteins from the biological environment to mediate binding to cell-specific epitopes. Lastly, the chemistry of the delivery vehicle can be modified to bind directly to epitopes on the target cell. Schematic not to scale.

Molecular targeting strategies complement administration methods by specifying the cell types that can interact with or take up genome editors. Most frequently, researchers engineer genome editors or delivery vehicles to bind to unique epitopes on the target cell. These non-covalent interactions are intended to drive uptake exclusively into the target cell, while sparing bystander cells (Fig. 2). Targeting motifs include antibodies and their derivatives, aptamers, fusogens, capsid proteins and metabolites.

Examples of targeted therapies in clinical trials include VERVE-201.^44–46^ VERVE-201 is an investigational gene editing therapy that uses *N*-acetylgalactosamine (GalNAc) - conjugated LNPs to deliver CRISPR base editors to the liver and inactivate the ANGPTL3 gene for treating homozygous familial hypercholesterolemia. Conjugation of LNPs with GalNAc allows them to enter cells using the asialoglycoprotein receptor pathway, which is highly expressed in the liver but not in other tissues. GalNAc-LNPs enable efficient delivery to the liver even in patients with homozygous familial hypercholesterolemia, who may have impaired low-density lipoprotein receptor activity that reduces the uptake of unconjugated LNPs.^45^ Similarly, the AAV capsids tested in clinical trials are chosen on the basis of tropism. Tissue tropism reflects the specific interactions between the capsid structure with cellular glycans and receptors.^47^ EPI-321 uses AAVrh74 to halt the expression of DUX4 and reduce muscle cell death in facioscapulohumeral muscular dystrophy (FSHD).^20^ AAVrh74 was first identified in rhesus macaques and has demonstrated widespread transgene delivery to skeletal, diaphragm, and cardiac muscle following intravenous administration, but the exact receptor used by AAVrh74 is unknown.^48^

Outside of clinical trials, researchers have attached targeting moieties directly to Cas9 and a variety of delivery vehicles. This rational design strategy requires the target cell of interest to have a known and often unique cell surface epitope. Antibodies can similarly be expressed or conjugated to the surface of delivery vehicles.^49^ The expression of single-chain antibody fragments on the surface of Enveloped Delivery Vehicles (EDVs), a virally-derived vesicle packaging Cas9 ribonucleoproteins, allowed uptake and genome editing only into cells with the corresponding surface antigen.^50–53^ This approach allowed genome editing of human T cells in an immunocompromised mouse.^50^ The conjugation of anti-CD117 antibodies onto the surface of LNPs allowed correction of sickle cell disease *ex vivo* in patient-derived blood cells, but was not tested *in vivo*.^54^ Other groups have displayed anti-CD45^55^, anti-EGFR^42^, and anti-CD52^56^ on LNP surfaces for genome editing applications.

While active targeting strategies use specific ligands on the delivery surface (e.g., antibodies, aptamers, peptides, etc.) to bind cell-specific receptors and promote selective uptake, passive targeting relies on the inherent material properties of the material (e.g., size, surface charge, lipid composition, etc.) to influence where particles accumulate in the body. For example, adding cationic lipids to LNPs could enrich them in the lung after intravenous administration, whereas neutral LNPs are enriched in the liver.^57–59^ While the mechanism of this change in enrichment is not fully understood, one hypothesis suggests that a cationic surface leads to the different adsorption of blood plasma proteins compared to a neutral surface, where the different sets of adsorbed proteins act as ligands binding to lung cell receptors and mediating uptake.^57^ Alternate hypotheses suggest that expression differences do not result from differences in biodistribution, but altered LNP intracellular processing and cargo expression in lung compared to liver cells.^60^

Researchers have also used barcoded screens of material compositions to identify designs with inherent tropism for specific receptors, cells and tissues. In these screens, designs are barcoded with oligonucleotides, such that the sequence of the barcode allows unambiguous identification of the design. Designs are then pooled together and administered into a model system. Target cells are then extracted, barcodes isolated, sequenced, and used to identify designs that are enriched within the cell. Multiple rounds of selection can be performed to improve tropism. This strategy has been used to evolve muscle-tropic AAVs (named “MyoAAVs”) with arginylglycylaspartic acid motifs^61^ and AAVs binding the transferrin receptor for transcytosis across the blood-brain barrier to enter the central nervous system.^62,63^ While initially applied to discover new AAV capsids^64^, this strategy has now been applied to LNPs.^65,66^ These pool-based selection methods are useful for gradually narrowing the specificity of the delivery vehicle, and complement the rational design of molecular targeting strategies. We caution that identifying designs with physical targeting is only possible if physical components of the genome editors or delivery vehicles are directly quantified. Commonly used readouts, such as transgene expression or editor function, convolute the physical delivery of agents and delivery vehicles with downstream intracellular processing steps such as endosomal escape and gene expression.

A critical limitation of molecular targeting methods is that they do not prevent non-specific uptake pathways, such as macropinocytosis, utilized by clearance cells like Kupffer cells in the liver.^67,68^ This non-specific uptake may lead to persistent uptake of delivery vehicles administered intravenously in the liver despite targeting strategies.^50,54,69^ Molecular targeting also requires unique cell-surface epitopes or antigens, which narrows the range of targetable cell types. Most AAV serotypes use glycans and receptors to enter cells that are expressed on multiple cell types. Consequently, AAV serotypes have tropism towards multiple cell types, such as AAVrh.8 for smooth muscle, liver and central nervous system cells, which will require additional targeting mechanisms, such as controlling gene expression, to enhance specificity. Lastly, molecular targeting relies on interactions occurring at sub-nanometer length scales. This necessitates administration methods to position delivery vehicles or enzymes close to their target cells within the same compartment. Molecular targeting alone is unlikely to significantly enhance accumulation in tissues inaccessible by the chosen administration route. Given these constraints, molecular targeting should be considered an enrichment strategy, which increases the probability of accumulation at a target tissue or cell type, rather than a precision-guided system delivering cargo.

## Controlling the physical location of genome editors by expression

**Figure 3.**
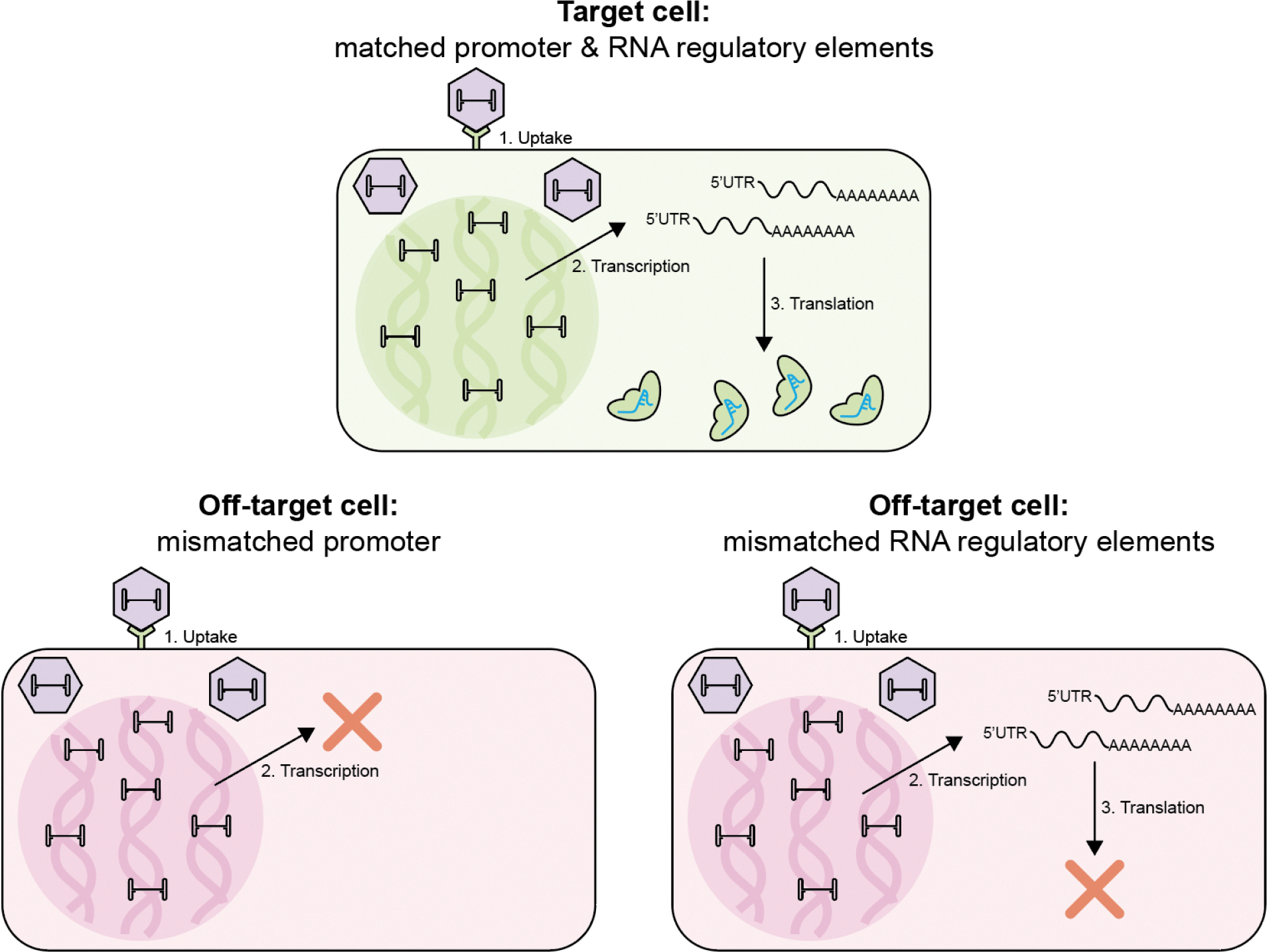
Schematic showing how the expression of genome editors can be controlled using promoters and RNA regulatory elements. In a target cell where the promoters and RNA regulatory elements match, transcription and translation can occur, allowing genome editors to be produced. In off-target cells, the promoter may not be recognized, preventing transcription. Alternatively, RNA regulatory elements may prevent the translation of mRNA in the off-target cells. Schematic not to scale.

Genome editors are composed of protein or nucleic acid components. Consequently, genome editors can be delivered as protein complexes or as their encoding nucleic acids. The delivery of genome editors-encoding nucleic acids could provide an additional level of specificity by controlling the types of cells that can transcribe or translate the nucleic acids into functional genome editors. This could ensure that the genome editors are only present in a specific subset of all the cells that received delivery vehicles (Fig. 3). One approach used in clinical trials to control the expression of genome editors is to use tissue-specific promoters. The promoter region of a cargo gene is the sequence involved in recruiting RNA polymerase to initiate transcription. Initiating transcription requires many transcription factors to assemble at the promoter region with the RNA polymerase. Each regulatory protein contributes to the control of expression, and some regulatory proteins are only found in specific cell types or cell states, which allows for cargo expression only in cells with these regulatory elements. For example, the AAV in EPI-321 uses the muscle-specific CK8e promoter to express the epigenetic editor.^70^ Similarly, HuidaGenes uses the neuronal-specific MECP2 promoter to express high-fidelity Cas13Y from their AAV to treat MECP2 duplication syndrome.^35,36^ Other cell specific promoters used preclinically include the CK8 promoter for muscle cells^71^, LP1 and LCAT promoters for liver^72^, cTnT promoter for heart^73^, and SYNI promoter for neurons^74^.

Control of transgene expression has also been explored at the level of translation using microRNA (miRNA)-based regulatory elements. In one implementation, the transgene mRNA is designed with binding sites for a miRNA that is abundant in off-target cells, such that the cell’s endogenous miRNA will bind to the transgene transcript and trigger its degradation. This “off-switch” approach has been applied to destabilize the AAV transgene in hepatocytes. miR122 is an abundant miRNA expressed specifically in the liver. miR122 target sequences were incorporated into the 3’-untranslated regions of an AAV9 SaCas9 vector. The authors found that incorporating the target sequences reduced leaky hepatic gene editing by >80%, without affecting on-target expression in cardiomyocytes.^75^ Tissue specific miRNA expression can also allow editing in specific tissues using miRNA binding sites to inhibit the translation of co-delivered anti-CRISPR proteins.^76–78^ Similarly, miRNA binding sites can be used to generate functional sgRNAs only in the presence miRNA enabling editing in specific cells.^79,80^ Other strategies to conditionally control sgRNAs include using ribozyme and toehold switches.^81^ Yet another strategy uses RNA editing enzymes called ADARs (adenosine deaminases acting on RNA) to control when an mRNA cargo is translated. The transgene mRNA is designed with a sensor sequence and premature UAG stop codons upstream of the transgene. The binding of specific endogenous RNA sequences in the target cells to the sensor sequence helps to recruit ADAR enzymes, which edit the adenosine in the stop codons to inosine, converting the stop codons into sense codons. This allows translation of the downstream transgene.^82–84^

Promoters and regulatory elements have limitations. Tissue-specific promoters often compromise expression strength for specificity, resulting in lower overall transgene expression.^85^ RNA-based regulatory elements frequently achieve only partial repression, with effectiveness dependent on variable endogenous RNA levels. Furthermore, we note that it is essential not to confuse the specific expression of cargo with the overall physical biodistribution of delivery vehicles. Although cargo expression may correlate with the physical distribution of delivery vehicles, these two parameters do not precisely overlap. Differences in intracellular trafficking, endosomal escape efficiency, nuclear entry, promoter compatibility, and innate immune sensing impact the capacity of various cell types to express foreign genes leading to mismatches between cargo expression and distribution.^69^ Delivery strategies relying solely on tissue-specific expression may still require relatively high systemic doses (or repetitive doses) to achieve a therapeutic effect, since delivery vehicles also reach non-target tissues diluting the effective dose. These approaches are unlikely to fully mitigate immunogenicity or toxicity, which are often driven by the location of the physical components of the delivery vehicles.

## Controlling where genome editors have a phenotypic effect

**Figure 4.**
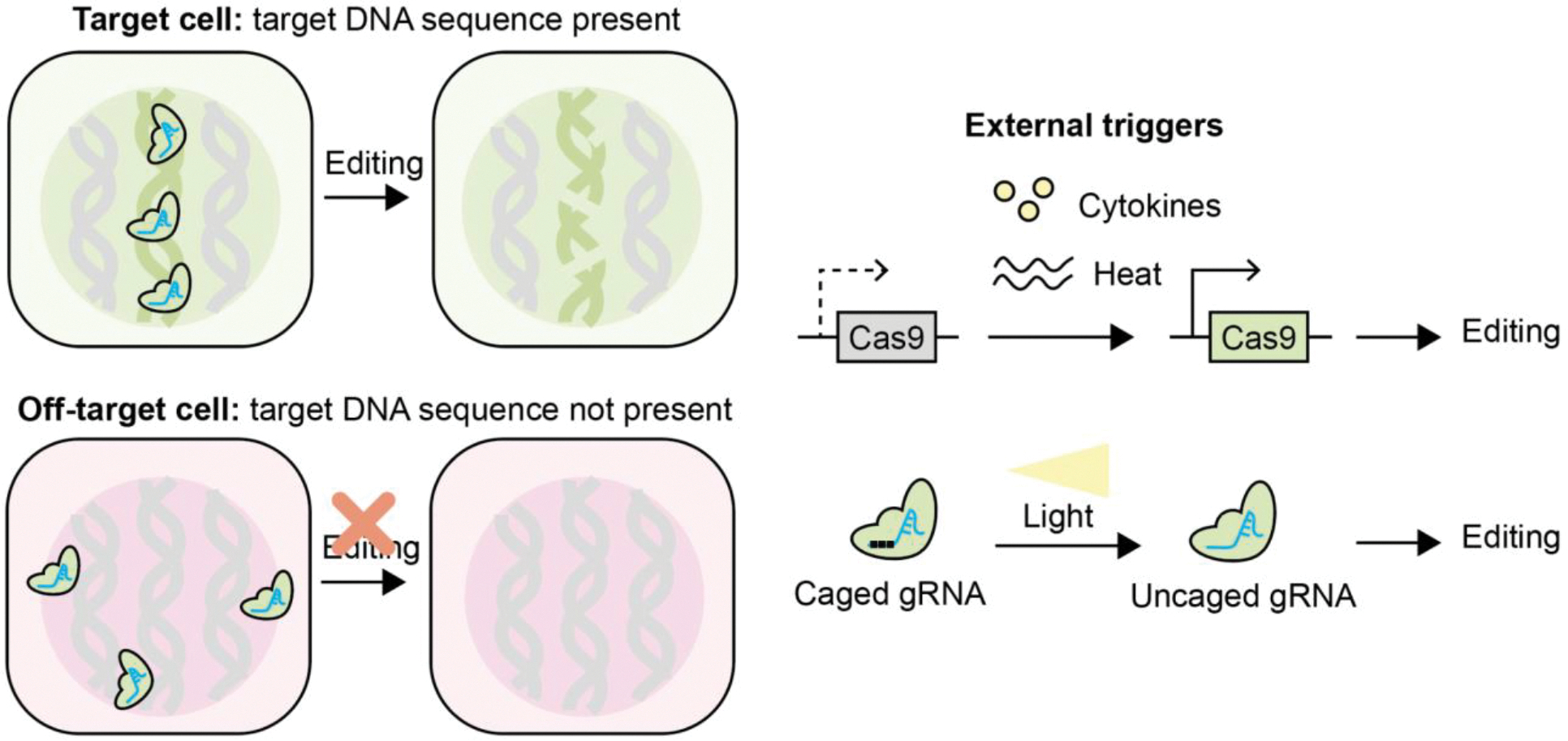
Schematic showing how the activity of genome editors can be controlled by the presence of target genetic sequences and external cues. In a target cell where the targeted genetic sequence is present, genome editors have a substrate to cut, and editing occurs. In off-target cells where the targeted genetic sequence is not present, even though genome editors are available, they have no suitable substrate. Alternatively, the expression and activity of either the genome editor or the guide RNA can be externally triggered through the use of small molecules, light, and heat sources to achieve spatiotemporal control of editing outcomes. Schematic not to scale.

As a last line of specificity, researchers can try to limit the activity of the enzymes to specific cells by carefully choosing target genetic sequences or incorporating conditional control mechanisms. Although every nucleated somatic cell contains the entire human genome, only a subset of genes is actively expressed in any given cell type. The pattern of gene expression varies widely between different cell types which gives rise to their specialized functions. Because not all genes are active in every cell, the functional effect of genome editing does not apply uniformly to every edited cell (Fig. 4). Gene editing therapies in clinical settings are predominantly focused on genes with distinct tissue- or cell-type specificity to enable precise interventions. For example, liver-specific genes, including PCSK9, are targeted in hepatocytes to address cardiovascular and lipid disorders, ensuring that the phenotypes effects of genome editing are largely limited to the liver even if there may be genome editors and editing in other organs.^9^ Likewise, BEAM-302 uses base editing to correct a restore the function of the *SERPINA1* gene, which encodes for alpha-1-antitrypsin. Alpha-1-antitrypsin is primarily produced by the liver.^12^

Genome editing can also be restricted to specific disease cells by exploiting their unique genetic sequences and relying solely on the specificity of the genome editor. Excision BioTherapeutics’ EBT-101 uses AAV packaging Cas9 to to excise integrated copies of type-1 human immunodeficiency virus.^19,86^ Non-infected cells would not be edited even if they received the AAV and the editing enzyme. EBT-101 met the primary and secondary endpoints of safety and biodistribution/ immunogenicity, but failed to prevent viral rebound in three patients that stopped anti-retroviral medication in a Phase 1/2 clinical trial.^87^ Preclinically, similar strategies have been used to knock out oncogenic mutations or fusion genes and correct mutant tumor suppressor gene. The goal is to kill malignant cells while leaving normal tissue unharmed. For example, gRNAs targeting specific repeat sequences in glioblastoma cells were designed to kill cells by inducing hundreds of double-stranded breaks.^88^ We caution that transporting genome editors to tumors in sufficient concentrations to illicit therapeutic effects remains challenging.

Conditional control of Cas9 activity has also been explored preclinically. Strategies developed to achieve this control include molecularly-,^89^ photothermally-,^90^ ultrasound-,^91^ and optically-induced activation of Cas9, which allows spatial and temporal regulation using external cues (Fig. 4). For example, an optogenetically activatable CRISPR-Cas9 nanosystem, termed nanoCRISPR, uses cationic polymer-coated gold nanorods to deliver a Cas9 plasmid under a heat-inducible promoter and enables precise genome editing controlled by near-infrared (NIR-II) photothermal activation in specific cells.^90^ In another instance, optical control of guide RNAs has been demonstrated by masking the spacer region with photolabile groups that can be removed through light-induced photolysis, enabling precise activation of gRNA-Cas9 complexes only upon illumination.^92^ Together, these advances provide versatile tools for finely tuning Cas9 activity in response to external stimuli, thereby minimizing off-target effects and improving editing specificity.

It may be challenging to identify targetable genetic regions that are sufficiently specific to allow perfect discrimination of healthy and diseased cells, especially if mutations differ by single base pairs. Single-base pair differences are sometimes tolerated within Cas9’s off-target recognition.^93^ Conditional control mechanisms may exhibit a low baseline level of editing. Adding control mechanisms increases the complexity of the enzyme and delivery vehicles could have unintended side-effects which may negatively impact its translation to patients. Ultimately, controlling where genome editors have a phenotypic effect is best used as the last line of specificity. These strategies do not explicitly control where enzymes are physically located and cannot mitigate risks associated with the accumulation of enzymes and delivery vehicles in unintended organs and tissues. They should be used in combination with different control strategies to ensure safe and adequate delivery of editing enzymes to the target site.

## Perspective

The rapid advancement of genome editors has outpaced our ability to deliver them safely and effectively to specific cells in the body. While delivery remains a persistent challenge, existing technologies have enabled the first wave of clinical successes, particularly in *ex vivo* therapies and metabolic liver diseases. However, despite these landmark achievements, there are concerns over the scalability and profitability of such therapeutics. We believe that unlocking safe and effective *in vivo* genome editing will reinvigorate the field, as it bypasses costly and time-consuming therapeutic cell manufacturing, while enabling treatment of a broader range of diseases. Continued advances in delivery will broaden the scope of treatable diseases and cell types. Improvements in targeting the lung, hematopoietic stem cells, and immune cells are likely to define the next wave of genome editing therapies. Importantly, precise physical targeting (i.e., genome editors and delivery vehicles only accumulate in target cells and tissues) may not always be necessary, if sufficient enzyme reaches the intended tissue to drive therapeutic benefit without incurring off-target toxicity, that may suffice clinically. To help determine what is sufficient and guide delivery strategies, it may be useful to quantitatively determine pharmacology parameters of genome editors. Useful parameters include efficacy (the maximum effect a drug can produce), EC50 (the effective concentration of a drug producing a 50% maximal response), and selectivity (the drugs ability to interact with the intended target while minimizing off-targets). These numbers could help researchers determine how many editors need to be delivered per cell.

To date, we have been successful in specifically delivering enzymes and their delivery vehicles to a few liver cell types. Achieving similar targeting in other tissues and cells remains an aspirational benchmark. Definitively demonstrating that genome editors and delivery vehicles are only physically present within a target site will require tracking both the components of genome editors and delivery vehicles *in vivo* across multiple timepoints to capture biodistribution and pharmacokinetics. This is an experimentally challenging endeavor. Standard methods relying solely on transgene expression or genome editing efficiency are inadequate for confirming where enzymes and delivery vehicles accumulate, as these metrics can vary widely between cell types due to differential protein expression and editing susceptibility. Similarly, single-timepoint measurements cannot account for the distinct degradation kinetics of enzymes and delivery components across different cell types. New developments and claims in targeted delivery must be carefully scrutinized to understand their level of specificity and precision.

**Figure 5.**
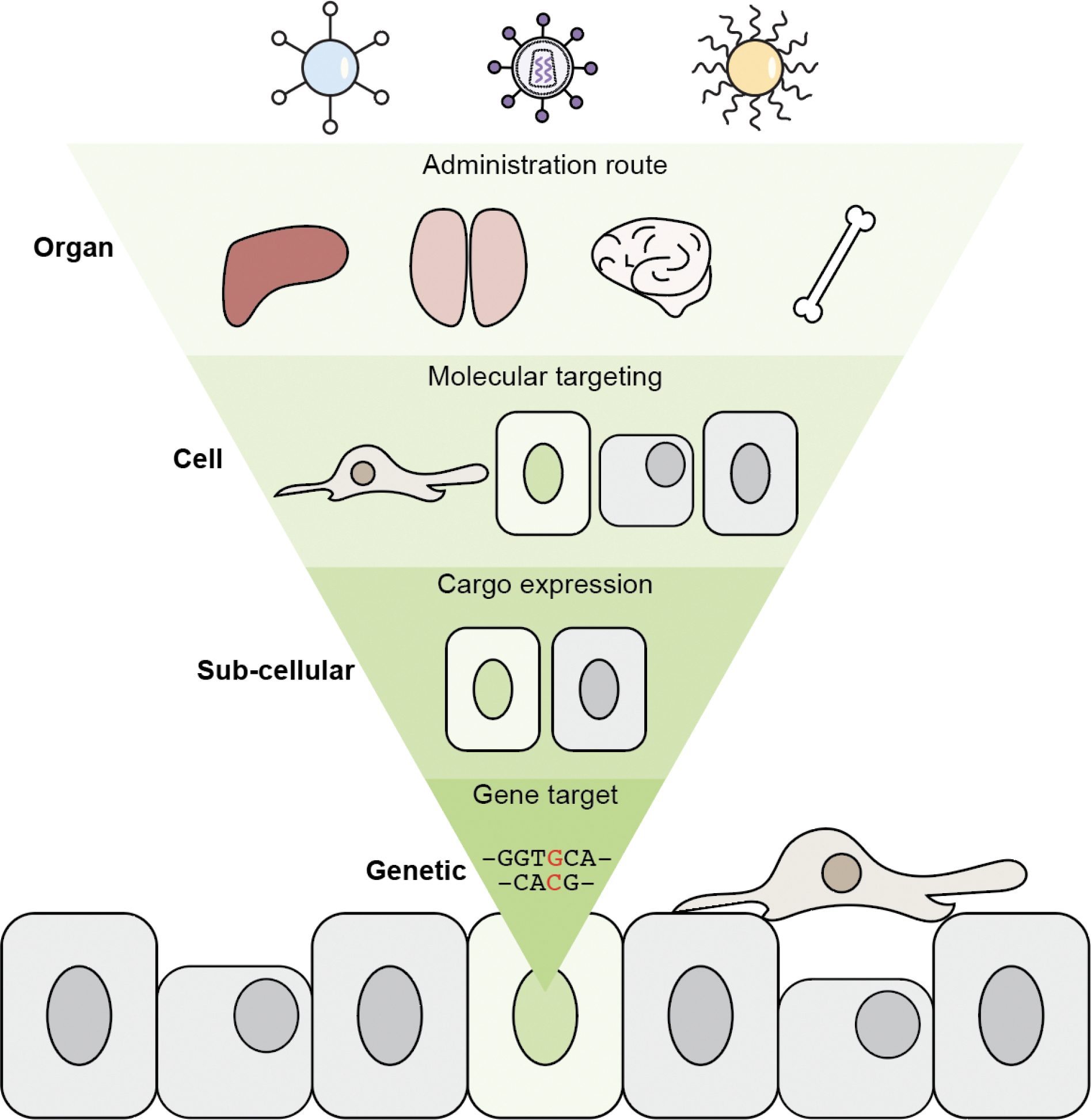
Specificity of genome editors and delivery vehicles can be increased by controlling specificity at multiple stages through the delivery process. Targeting can be thought of as a series of selection filters that progressively narrow which organs and cells are affected by a genome editor. Ideally, delivery vehicles should be chosen to narrow down delivery at the organ, cell, subcellular and genetic level. Each mechanism acts as a layer of specificity that narrows the distribution and activity of genome editors to the desired cells. Schematic not to scale.

The future lies in designing delivery platforms that control the specificity of genome editors and delivery vehicles at multiple stages through the delivery process (Fig. 5). We note that a number of clinically successful strategies already combine two or more specificity mechanisms. We propose formally conceptualizing targeting as using a series of selection filters that progressively restrict specificity. Each mechanism acts as a layer of specificity that narrows the distribution and activity of genome editors to the desired cells. These mechanisms include physical targeting through administration, cellular uptake via receptor-ligand interactions, cell-type-specific expression using transcriptional or translation control elements, and choice of gene target. Once a disease indication is selected, researchers can refine and optimize each layer from administration to choice of gene target independently. This approach acknowledges that no single mechanism is likely to be sufficient to achieve absolute specificity, particularly across diverse tissues or disease states. Instead, by stacking multiple mechanisms, the system can progressively eliminate off-target delivery and activity. While it may not be feasible to implement all layers in every therapeutic context, we believe this framework provides a useful conceptual guide for rationally designing delivery systems. It encourages the integration of complementary and synergistic targeting strategies to increase precision, reduce off-target effects, and improve safety.

Controlling specificity at multiple stages of delivery will also require a thorough understanding of the delivery mechanism of administered editors. This will require a deep mechanistic understanding of where genome editors and delivery vehicles go, where they are expressed, and where they are functionally active. Advances in non-invasive, quantitative imaging tools like PET and multimodal reporters will be essential for real-time assessment of biodistribution and editing activity. These datasets can then be integrated with machine learning models to predict, optimize, and iterate delivery outcomes.

We envision a future where versatile delivery platforms are available for nearly any disease target, enabling genome editors and targets to be seamlessly exchanged to meet therapeutic needs. To build this future, extensive collaborations will be required between biochemists, molecular biologists, cell biologists, biomedical engineers, chemists, material scientists, chemists, clinicians and investors. The complexity of the body requires that we all work on building “magic bullets” together.

## Figures and Tables

**Box 1 :** Summary of major delivery modalities for genome editors.

Delivery modality	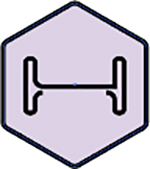	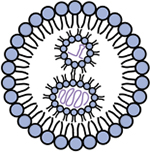	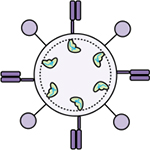	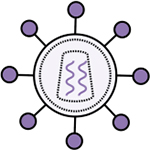	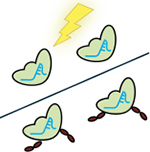
	AAV	LNP	EDV/ VLP	Retroviruses	RNP
**Safety**	Low risk of genome integration^94^, unless combined with nucleases and used as a homology directed repair template.^95,96^ Possibility of pre-existing immunity^97,98^	No reported risk of genome integration Low possibility of pre-existing immunity^99^	No reported risk of genome integration Low possibility of pre-existing immunity^100^	Random gene integration^100^ Low possibility of pre-existing immunity^100^	No reported risk of genome integration Possibility of pre-existing immunity^101^
**Dosing**	Repeat dosing prevented by adaptive immunity to capsid^102^	Repeat dosing possible, but adaptive immunity to transgene protein is a risk^103,104^	Repeat dosing likely prevented by adaptive immunity to surface proteins^105^	Repeat dosing prevented by adaptive immunity to surface proteins^105^	Used ex vivo; cell-penetrating peptides and other modifications necessary for in vivo use^106,107^ Repeat dosing likely prevented by adaptive immunity to protein^108^
**Manufacturing**	Mammalian / insect cells^109^ Frozen for storage^110^	Synthetic^104^ Lyophilized for storage^111^, may also be frozen^112^	Mammalian cells^2,50,51,113^ Frozen for storage	Mammalian cells^114^ Frozen for storage^115^	*E.coli* / cell-free expression Frozen for storage
**Cargo**	4 – 5 kb DNA ( stable transgene expression from episome)^116^	RNA, DNA and ribonucleoprotein demonstrated^59,104,117^	Ribonucleoproteins or mRNA with guide RNA^50–52,118–121^	<10 kb RNA that is reverse transcribed and integrated into the recipient cell^100^	Ribonucleoprotein
**Targeting mechanisms**	Natural and engineered capsid serotypes for molecular targeting^64,122^ Capsid conjugation with targeting motifs for molecular targeting^123^ Cell-specific promoter to control which cells express transgene^124^	Surface conjugation with targeting motifs for molecular targeting^54,55,125,126^ Targeting motifs can also be inserted after particle formation (using methods such as ASSET).^42,127^	Targeting motifs expressed on the surface for molecular targeting^50^ Conjugation of targeting motifs for molecular targeting^49^	Targeting motifs expressed on the surface for molecular targeting^128,129^ Conjugation of targeting motifs for molecular targeting^49^ Cell-specific promoter to control which cells express transgene	Conjugation with targeting motifs for molecular targeting^130^ Gene target can be chosen to control where functional effects are present Domains can be added to control where the enzyme is functional conditionally
